# Novel Strategy to Improve the Performance of Localization in WSN

**DOI:** 10.1155/2015/149767

**Published:** 2015-09-07

**Authors:** M. Vasim Babu, A. V. Ramprasad

**Affiliations:** ^1^Department of Electronics and Communication Engineering, Latha Mathavan Engineering College, Madurai, Tamil Nadu 625301, India; ^2^Department of Electronics and Communication Engineering, K.L.N. College of Engineering, Sivagangai District, Pottapalayam, Tamil Nadu 630612, India

## Abstract

A novel strategy of discrete energy consumption model for WSN based on quasi Monte Carlo and crude Monte Carlo method is developed. In our model the discrete hidden Markov process plays a major role in analyzing the node location in heterogeneous media. In this energy consumption model we use both static and dynamic sensor nodes to monitor the optimized energy of all sensor nodes in which every sensor state can be considered as the dynamic Bayesian network. By using this method the power is assigned in terms of dynamic manner to each sensor over discrete time steps to control the graphical structure of our network. The simulation and experiment result shows that our proposed methods are better in terms of localization accuracy and possess minimum computational time over existing localization method.

## 1. Introduction and Related Work

In many real time applications of wireless senor network, localization [1–3] plays an important parameter to identify the location of an object or moving stimuli in geographical area. But still there are some research challenges available in order to improve the localization accuracy [4], better energy consumption model, and reduce the localization error while finding the moving stimuli. Various localization algorithms and analytical models have been proposed [5–7] for past decades based on centralized, distributed [8, 9], bacon based, diffusion based, and bounding box localization algorithms. But these algorithms may either suffer from low energy consumption or poor sampling efficiency. Particularly in GPS based localization method [10] the line of sight problem is a major issue and the power consumption in GPS will reduce the entire battery life of the wireless sensor network.

In order to minimize the cost of energy in GPS model, few nodes which are considered as beacon nodes represent the GPS modules. As the geographical area increases, the number of beacon nodes also increases which leads to high cost. Another popular localization method which has wide range of possible applications is called source localization method. In this method author has analyzed both indoor and outdoor applications [11] including movement of vehicle and also tracked the human voice.

There are so many ways to implement the source localization in real time environment based on energy, AOA, and TDOA which are the high level parameter of WSNT. The further classification of source localization [12] is single and multiple target localization in WSN and WBSN. However, very few papers are investigated for the purpose of multiple target localization scheme. All these papers are based on the maximum likelihood estimation. The RSS of this method could be calculated in the following manner:

(1) $$\begin{array}{c} Y_{i}\left(t\right)=h_{i}\overset{L}{\underset{l=1}{\sum }}\frac{A_{l}\left(t\right)}{d_{il}^{k}\left(t\right)}+\varepsilon_{i}\left(t\right), \end{array}$$

where *d*_*il*_^*k*^ is the distance between the *i*th sensor and the *L*th source and *h*_*i*_ is the gain of the *i*th sensor.

Range based [13] and range-free localization technique [14, 15] are under the self-localization method. The classical method of range based localization is used to estimate TOA [16], TDOA, RSSI [17], and AOA and the range-free method is used in pattern matching and hop count based applications.

In this paper we proposed a new scheme of localization method based on quasi and crude Monte Carlo technique. In our energy consumption model we consider novel discrete generalization of hidden Markov model to balance the node energy within the particular samples of dynamic Bayesian networks. In our sensor model we assume that each sensor power acts independently and cost of each sensor consists of weighted discrete power storage from supervisor node. The power update is based on the number of hop counts from sender to receiver at a particular state.

The rest of the paper is organized as follows.  Section 2 briefly discusses the network model of Bayesian network in discrete manner.  Section 3 elaborates the crude Monte Carlo method analysis in three-dimensional manner. The details of discrete power monitoring strategy are discussed in Section 4. We evaluate the performance of proposed method discussed in Section 5.

## 2. Proposed Method

### 2.1. Network Model

Our network model is based on Bayesian network. In this model we assume that each sensor power acts independently and cost of each sensor consists of a weighted amount of discrete power storage. The power update is based on the number of hop counts from sender to receiver at a particular state.

The behavior of discrete time dynamic system is described by

(2) $$\begin{array}{c} x\left(k+1\right)=f\left(x\left[k\right],u\left[k\right],k\right). \end{array}$$

Let us assume that the sensor observation time is *t* by the state variable *y*_*t*_ with probability function *p*(*x*) with hidden Markov model. It can be denoted by state equation and by the following observation:

(3) $$\begin{array}{c} \text{State equation}⟶x_{t}\sim q_{t}\left(\cdot \;|\;x_{t-1},\theta \right),\\ \text{Observation equation}⟶y_{t}\sim f_{t}\left(\cdot \;|\;x_{t},\varphi \right), \end{array}$$

where *y*_*t*_ is the observations of the time of the packets arriving sequentially and *x*_*t*_ is the state variables of interest which is the current posterior distribution of *x*_*t*_:

(4) $$\begin{array}{c} \Pi_{t}\left(x_{t}\right)⟶\int q_{t}\left(x_{t}\;|\;x_{t-1}\right)f_{t}\left(y_{t}\;|\;x_{t}\right)\pi_{t-1}\left(x_{t-1}\right)dx_{t-1}, \end{array}$$

where *a* is the integer factor to count the total number of states in entire sensor network from one hop to another.

Choose an integer *a* ∈ {1,2,…, *k* − 1} and let

(5) $$\begin{array}{c} P\left(\theta \;|\;x\right)=\left\{\frac{i-1}{k}\left(1,a,\ldots ,a^{r-1}\right)\mathrm{mod}1,\;\;i=1,\ldots ,k\right\}. \end{array}$$

*P*(*θ*∣*x*) is the posterior probability that was observed as the function of the unknown model parameter like RSS of each sensor hop in the network. The current state *y*_*t*_ is independent of all the states prior to *T* − 1. In this communication model we take three centralized discrete nodes. Each centralized node has three subordinate nodes to communicate with each other based on Bayesian network model. Let *A*_1_, *A*_2_, and *A*_3_ be three centralized nodes which are going to share the power to their neighboring nodes with the help of Bayesian network.

**Figure 1:**
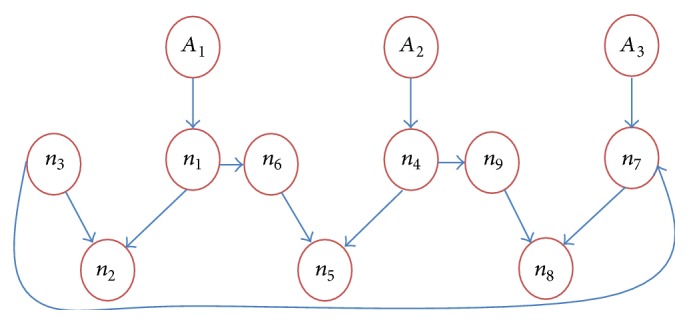
Bayesian model.

Figure 1 shows that the three independent nodes *A*_1_, *A*_2_, and *A*_3_ (three states) are connected with the subnodes *n*_1_ to *n*_8_. Each centralized node can potentially depend on other nodes, that is,

(6) $$\begin{array}{c} A_{1}\text{ connected with }n_{1},n_{2},n_{3},\\ A_{2}\text{ connected with }n_{4},n_{5},n_{6},\\ A_{3}\text{ connected with }n_{7},n_{8},n_{9}. \end{array}$$

The conditional probabilities of above Bayesian network are described by the following factorization:

(7) $$\begin{array}{c} P\left(A_{1},n_{1},n_{2},n_{3}\right)=P\left(A_{1}\right)P\left(n_{1}\right)P\left(n_{2}\;|\;A_{1}\right)P\left(n_{3}\;|\;n_{1}n_{2}\right)=P\left(A_{1}\;|\;n_{2}\right)P\left(n_{3}\;|\;n_{1}n_{2}\right),\\ P\left(A_{2},n_{4},n_{5},n_{6}\right)=P\left(A_{2}\right)P\left(n_{4}\right)P\left(n_{5}\;|\;A_{2}\right)P\left(n_{6}\;|\;n_{4}n_{5}\right)=P\left(A_{2}\;|\;n_{5}\right)P\left(n_{6}\;|\;n_{4}n_{5}\right),\\ P\left(A_{3},n_{7},n_{8},n_{9}\right)=P\left(A_{3}\right)P\left(n_{7}\right)P\left(n_{8}\;|\;A_{3}\right)P\left(n_{9}\;|\;n_{7}n_{8}\right)=P\left(A_{3}\;|\;n_{8}\right)P\left(n_{9}\;|\;n_{7}n_{8}\right). \end{array}$$

This factorization shows a set of conditional independence relations of each node. So dynamically all the nodes of Bayesian network are interconnected in time series modeling. By using this time series method we can predict the time index “*t*” to each independent node like *a*_1_, *a*_2_, *a*_3_ for a sequence of data {*Y*_1_,…, *Y*_*T*_. Each state is directly influenced by the previous state; that is,

(8) $$\begin{array}{c} P\left(Y_{1},Y_{2},Y_{3},T\right)=P\left(Y_{1}\right)P\left(Y_{2}\;|\;Y_{1}\right)P\left(Y_{3}\;|\;Y_{2}Y_{1}\right),\ldots ,P\left(Y_{T}\;|\;Y_{T-1}\right). \end{array}$$

**Figure 2:**
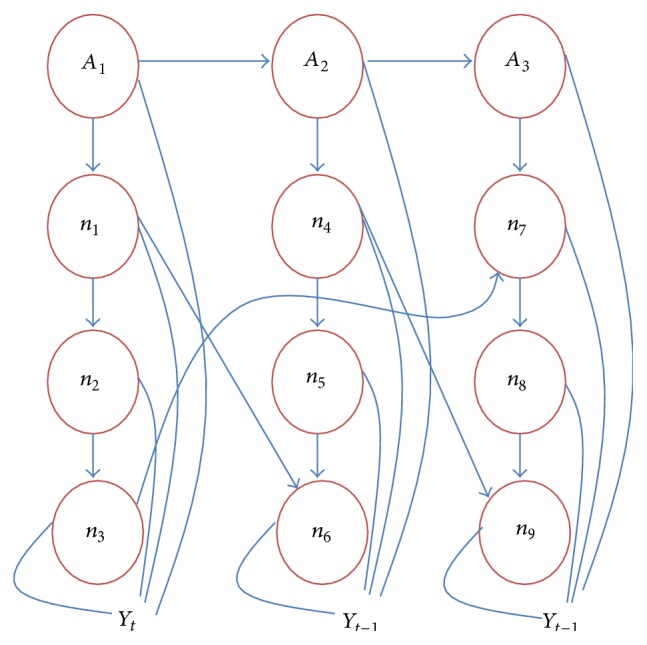
Markov model of Bayesian network.

Based on the above condition we can draw the conditional independence relations between states which is shown in Figure 2. This Markov relation model extends the static modeling to dynamic Bayesian model in discrete manner.

## 3. Efficient Crude Monte Carlo 3D Analysis

In this section we introduce the crude Monte Carlo method to analyze the sensor behavior of each iteration. In this method we select the number of wireless sensors, say, *x*, *y*, *z* moving randomly from the interval [0, *T*_*i*_] and the approximate sample integral by

(9) $$\begin{array}{c} I_{\text{crude}}=\overset{T_{i}}{\underset{0}{\int }}f\left(x,y,z\right)dx\;dy\;dz\\ \approx \frac{1}{N_{i}}\overset{N-1}{\underset{i=1}{\sum }}f\left(x_{i},y_{i},z_{i}\right). \end{array}$$

The average number of *N* samples can be calculated from *f*(*x*_1_, *x*_2_, *x*_3_), *f*(*y*_1_, *y*_2_, *y*_3_), and *f*(*z*_1_, *z*_2_, *z*_3_). Then we estimate the overall coverage of assigned sensor to find the interval between *N* number of sensors. For example, the statistical interval between *a* and *b* can be calculated by

(10) $$\begin{array}{c} {\text{crude}}_{T_{i}}=\langle b-a\rangle \langle a-c\rangle f\left(x,y,z\right)=\langle b-a\rangle \langle a-c\rangle \frac{1}{N_{i}}\overset{N-1}{\underset{i=1}{\sum }}f\left(x_{i},y_{i},z_{i}\right), \end{array}$$

where *x*_*i*_, *y*_*i*_, *z*_*i*_ is a random dimension in the geographical area within the unit cube *x*, *y*, *z*∈[0, *T*_*i*_]. So the interval calculation for this three-dimensional axis is to generate the *N* number of sensors; that is,

(11) $$\begin{array}{c} {\text{crude}}_{\text{3DAxis}}=\langle a_{2}-a_{1}\rangle \langle b_{2}-b_{1}\rangle \langle c_{2}-c_{1}\rangle \frac{1}{N_{i}}\overset{N-1}{\underset{i=1}{\sum }}f\left(x_{i},y_{i},z_{i}\right). \end{array}$$

To improve the accuracy of crude Monte Carlo we can use a priori knowledge from past samples that have the greatest impact on the positive weight function *w*(*x*). Consider a discrete time based power management system with state space.

*X*_*t*_ ∈ *X* and observation state is *y*_*t*_ ∈ *Y*. The Bayesian network system is indicated by the following prior states of data:

(12) $$\begin{array}{c} P\left(X_{0}\right)⟶\text{Initial power management distribution function},\\ P\left(X_{t+1}\;|\;X_{t}\right)⟶\text{Discrete power assignment transition law when }t=0,1,2,\ldots ,\\ P\left(Y_{t}\;|\;X_{t},Z_{t}\right)⟶\text{Overall power measurement of various state},\\ P\left(Z_{t-1}\;|\;X_{t},Y_{t}\right)⟶\text{Previous state measurement}. \end{array}$$

It is denoted as three-dimensional state variables:

(13) $$\begin{array}{c} X_{00:t}=\left\{x_{0},\ldots ,x_{t}\right\},\\ Y_{01:t}=\left\{y_{1},\ldots ,y_{t}\right\},\\ Z_{10:t}=\left\{z_{1},\ldots ,z_{t}\right\}. \end{array}$$

Now we can assume the hidden Markovian state property for power state variation in network; that is,

(14) $$\begin{array}{c} P\left(X_{t+1}\;|\;X_{00:t},y_{01:t}\right)=P\left(X_{t+1}\;|\;X_{t}\right),\\ P\left(y_{t}\;|\;X_{00:t},y_{01:t-1}\right)=P\left(y_{t}\;|\;x_{t}\right),\\ P\left(z_{t-1}\;|\;X_{10:t},y_{11:t+1}\right)=P\left(z_{t-1}\;|\;y_{t+1}\right). \end{array}$$

From the above equation we can specify the state equation of weighted samples as

(15) $$\begin{array}{c} X_{t}=f\left(x_{t},W_{t}\left(\text{Present weight state}\right)\right),\\ Y_{t-1}=f\left(x_{t}\;|\;y_{t},W_{t-1}\left(\text{Past weight state}\right)\right),\\ Z_{t+1}=f\left(z_{t}\;|\;x_{t},y_{t},W_{t+1}\left(\text{Future weight state}\right)\right). \end{array}$$

Then we compute the posterior state of the above hidden model for MMSE estimation:

(16) $$\begin{array}{c} {\hat{P}}_{t}\left(X_{t}\right)=P\left(X_{t}\;|\;y_{01:t},y_{t}\;|\;y_{10:t},z_{t-1}\;|\;y_{11,t}\right)\;x_{t},y_{t},z_{t}\in X,Y,Z. \end{array}$$

The state of Bayesian network can easily be extended to other types of network to represent the additional measurement of power variations. Already we have mentioned that the hidden Markov model is playing a major role in predicting the a priori probability of Bayesian network. So in this case Bayesian network is at the bottom level and hidden Markov model is at the top level of our design. So the joint probability of this Bayesian network can be expressed as the model of hidden Markov model. It is expressed as

(17) PZ,Y,X,Q=PZ ∣ X,Y,Q∗PX,Y ∣ Q∗PX ∣ Q∗PQ,

is the independent variable that measures the number of hidden states of each round. Our hidden Markov model can be viewed as a speculation of a mixture model, where the concealed variables which control the mixture part are to be chosen for every Markov transform as opposed to autonomous of one another.

## 4. Power Monitoring Strategy

During this period the energy of each iteration time is calculated and stored into its respective timeslot of present, past, and future time periods.

To monitor the energy of each sensor node in Bayesian network we take two clocking signals for power management system. Clock1 will synchronize all the power in entire system and allotted power after resampling. This is stored in Clock2. Both clock1 and clock2 have four bit periods to change lower to higher. Totally we have sixteen combinations of power states. This has been used for power mapping strategy. Each state has a unique discrete power from 0000 to 1111 as per the timeslots of clocking signals. For example, we consider the following power strategy.

The threshold RSS value of node 1 to node 3 is 4.2 units. But the packet information of node 1 is high compared to the threshold value. Now the corresponding state of node 1 will be changed to other states in terms of power. So we can achieve packet transmission along with power. It is shown on the following list:

 module clock1, clock2
 Clock1 C_1_[4]
 Clock1 C_2_[4]
 C_1_: [0001];
 C_2_: [1111];
 Power mapping()
 {
 Map1: 0000:C_1_:0001:C_2_
 Map2: 0001:C_1_:0010:C_2_
 Map3: 0010:C_1_:0011:C_2_
 Map4: 0011:C_1_:0100:C_2_
 MapN: 1110:C_1_:1111:C_2_
 }
 End timer.

For time synchronization of each node we have assigned four different timing periods (*T*_syn1_, *T*_syn2_, *T*_syn3_, *T*_syn4_) to monitor the sender and receiver time synchronization. Sender node *n*_1_ communicates something specific with its nearby time *T*_syn1_ as a timestamp, and receiver node *n*_2_ gets this information at its nearby time *T*_syn2_, where *T*_syn2_ = *T*_syn1_ + *d* + *ε*. Here, *d* and *ε* speak to the remaining nodes between the two nodes and intimate individually to other nodes. At time *T*_syn3_, node 2 sends back an affirmation packet. This information contains the qualities of *T*_syn2_ and *T*_syn3_, and node 1 gets the information at *T*_syn4_. So also, *T*_syn4_ is identified with *T*_syn3_ as *T*_syn4_ = *T*_syn3_ + *d* − sender node 1 can now measure the clock counterbalance and end-to-end postpone as *d* = [(*T*_syn2_ − *T*_syn1_)+(*T*_syn4_ − *T*_syn3_)]/2 and *d* = [(*T*_syn2_ − *T*_syn1_) − (*T*_syn4_ − *T*_syn3_)]/2. We have followed the following steps to allocate discrete power for entire network.

### 4.1. Discrete Markov Energy State Analysis

To amplify the system lifetime, the determination of a hand-off node needs to minimize the arrival at the midpoint of transmit power and parity of the utilization of battery vitality at distinctive transfers.

So the designated power is discrete and the starting battery energy at the transfers is limited, and the set of all residual energy levels is discrete and limited. The development of the remaining energy levels at all states can then be demonstrated as a limited state concealed Markov chain and the system lifetime is, thus, derived through the investigation on the hidden Markov chain.

**Figure 3:**
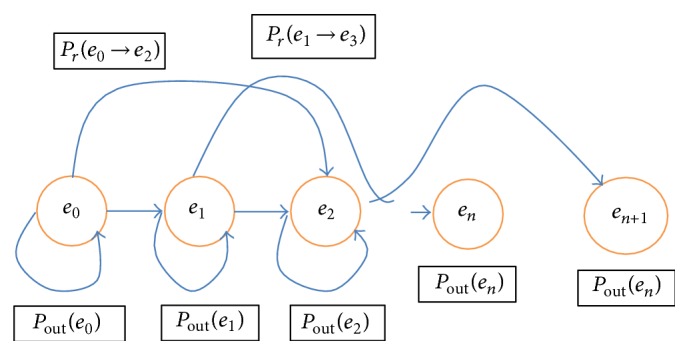
Energy state analysis.

The state space of the system, SP, is characterized as the function of all conceivable residual energy levels. The overall energy analysis of the system is a discrete hidden Markov chain, which can be informed to the state transition chart as indicated in Figure 3 that comprises all states and the move probabilities among states.

General steps to allocate discrete power in Bayesian network are as follows.

(1) Assign all the nodes randomly within Bayesian network space with location of three-dimensional axis {Loc_*xi*_, Loc_*yi*_, Loc_*zi*_} and discrete energy of sensor nodes *E*_0_, *E*_1_,…, *E*_*N*−1_.
(2) Then find the distance (transmission range) of each sensor in terms of discrete samples by using antithetic variates (variance reduction method).
(3) Based on the RSSI value, assign a discrete power to each sensor using crude Monte Carlo method.
(4) Then we perform each sensor node “*i*” in the Bayesian network that runs a number of iterations to check whether the RSSI value exceeds with allotted power of each sensor or not.
(5) Suppose the RSSI value is exceeded when compared with the predefined value; we update the location information and recalculate the needed discrete power of sensors with respect to RSSI value.
(6) Then resample the discrete power and mapping from centralized node to updated location of sensor.
(7) Within this time period we monitor each iteration and it is monitored by a timer *T*_*i*_ (present), *T*_*i*−1_ (past), and *T*_*i*+1_ (future) with the help of hidden Markov process.
(8) Finally it sends the data to destination path along with the location information and at last reset the timer.

## 5. Performance Evaluation

In this segment we analyze the performance of our proposed method with DQMCL and MCL based on localization method through simulation and experimental result. In simulation environment the sensor nodes were initially randomly distributed as per Figure 1 with Bayesian network where communication probabilities in Bayesian networks are assumed to follow Markov model. The following parameters are taken to analyze our method with the existing one:

- energy consumption,
- computational time,
- localization error,
- delay time,
- jitter analysis,
- estimation error based on localization steps,
- sensor lifetime,
- optimal time,
- overall performance.

### 5.1. Energy Consumption

**Figure 4:**
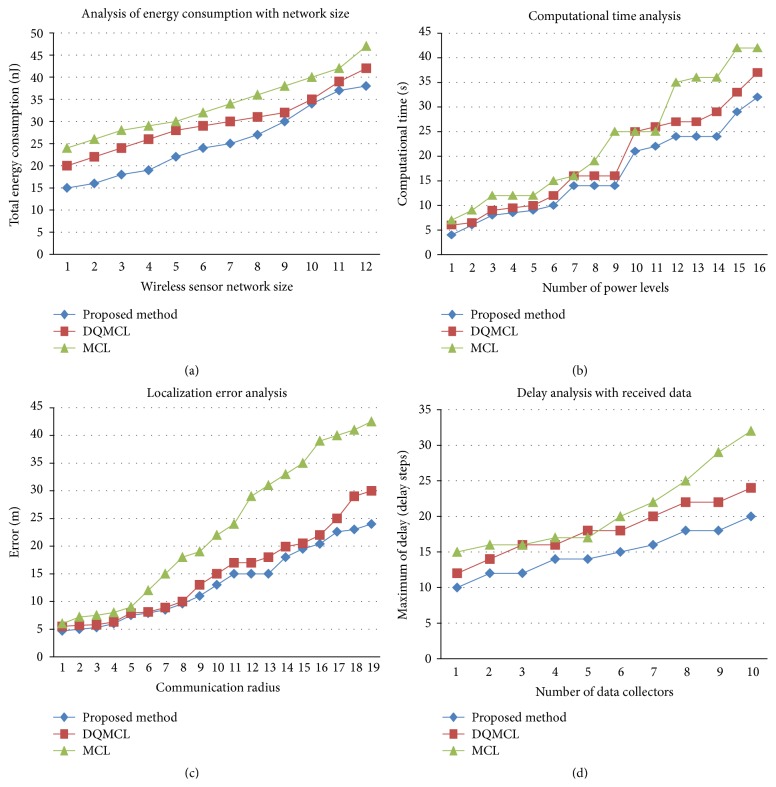


Figure 4(a) shows the comparison of total energy consumption based on Bayesian network size. It can be observed that our proposed method has taken a less energy of 37 nJ approximately to transmit the discrete packets.

### 5.2. Computational Time

Figure 4(b) shows the computational time of 32 seconds for mapping the power between various sensors consisting of 16 states of power compared to DQMCL and MCL method.

### 5.3. Localization Error

Figure 4(c) shows the localization error of our proposed method. It is less (maximum 19 m) compared to other two methods when we increase the communication radius.

### 5.4. Delay Time

Based on the number of data received from various states, Figure 4(d) shows the delay step is 20 for proposed method compared to delay steps of MCL and DQMCL of 32 and 24, respectively.

### 5.5. Jitter Analysis

**Figure 5:**
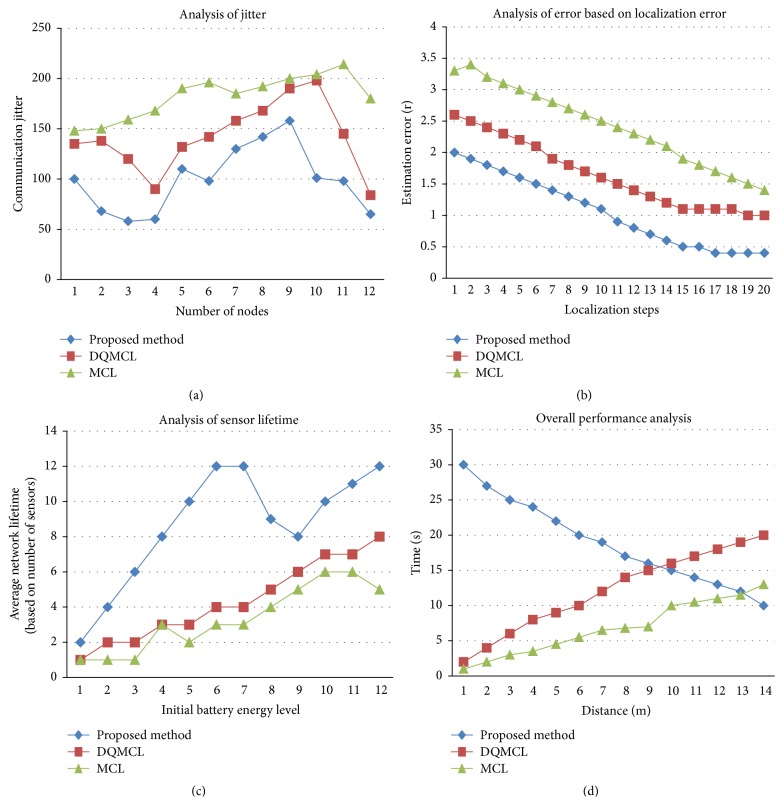


Figure 5(a) shows the average jitters of two algorithms along with proposed method. The proposed algorithm jitter is less (not exceeding the jitter range of 152), because of the reduced communication.

### 5.6. Estimation Error Based on Localization Steps

When we increase the localization step size, the estimation error is low (0.5 at 20th localization steps) compared to other two methods which are shown in Figure 5(b).

### 5.7. Sensor Lifetime

Figure 5(c) shows the overall sensor lifetime of various sensor energy levels for three algorithms. Our proposed method has maintained 12 sensors in active region with 6th, 7th, and 12th energy level compared to other two methods.

### 5.8. Overall Performance

Figure 5(d) shows the overall performance level of our proposed method along with analysis of optimal time, cluster overhead, and overall time for packet and power transmission. The optimal time of our method gradually reduces towards 10 sec at the distance of 14 m.

Table 1 shows the analysis report for overall computational time with standard threshold value for three methods. The proposed method has achieved low computational time to reach expected optimal threshold value compared to other two methods.

Table 2 shows the RSSI analysis of proposed method along with existing method. The variations of RSSI value of novel strategy are quite comfortable to compare with DQMCL and MCL based localization.

### 5.9. Experimental Result Analysis

In order to analyze the proposed method, an experiment is essential to verify the overall performance of proposed method. In this experiment we take CC2430 [19] radio chip to analyze the energy consumption model in terms of discrete power by using MICA2 motes with the help of tiny os which deals with low power wireless devices. The sensor node is placed as in Figure 1 by using Bayesian network. *A*_1_, *A*_2_, and *A*_3_ can act as centralized node and the rest of the nodes were considered as subordinate node to transmit and receive the information. For this experiment each sensor node has seven power levels based on CC2430 radio chip. When RSSI value exceeds the threshold value, the corresponding centralized node increases the power state and maps the required sample value. The power levels of each sensor have been compared frequently with power state to measure the RSS value.  Table 3 shows the power level of CC2430 [18] with power state. The discrete power levels of CC2430 can be tuned at the time of transmitted time which were monitored by power state of Bayesian network. CC2430 has excellent receiver sensitivity with low current consumption, so the overall energy consumption is quite comfortable for packet transmission in Bayesian network.

Through this setup we measure the distance between the located sensor node and corresponding RSSI value which were carried out.  Table 4 shows the RSSI value for various power levels along with transmission packet ratio with corresponding distance.

After analyzing the RSSI value nodes *A*_1_, *A*_2_, and *A*_3_ compare with threshold value and map the required sample value to corresponding sensor. In this experiment we measure the effect that the number of centralized nodes has on localization accuracy. Based upon the power transmission from centralized node to another we measure the overall accuracy of the proposed method.  Table 5 shows the accuracy of the proposed method compared with other two methods with the help of localization error.

**Figure 6:**
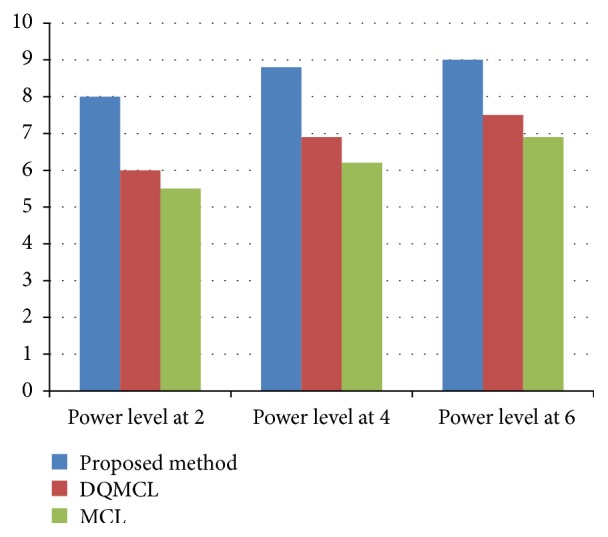
Packet transmission ratio at different power level.

Based upon the above measurement our proposed methods have minimum localization error and have good localization accuracy. In every iteration the energy of each sensor node has been monitored and maintains the RSSI value up to the level. This behavior is attributed to the number of localized nodes increasing with the number of iterations. When the number of iterations is increased, centralized node is quite balanced with respect to the RSSI value.  Table 5 clearly shows that our proposed method has low localization error compared to DQMCL and MCL based localization method. The energy levels of each iteration can be monitored by centralized node with respect to the predefined threshold value and analyze the overall structure of the Bayesian network.  Figure 6 shows the overall packet transmission ratio for proposed method with existing localization technique with different discrete power levels.

**Figure 7:**
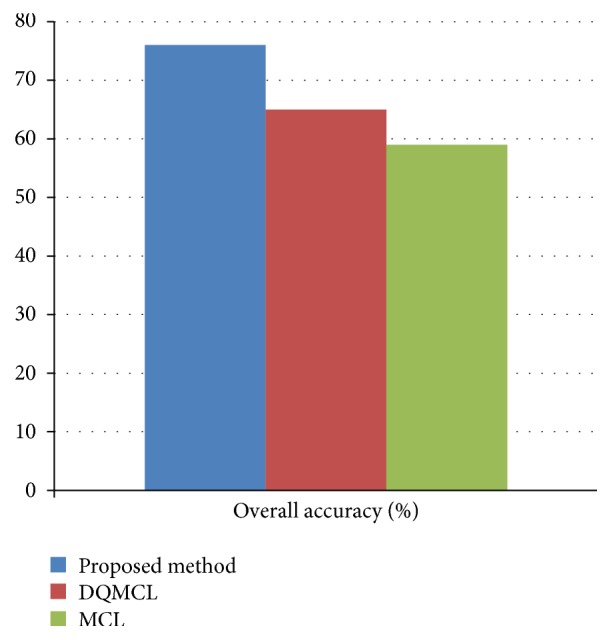
Overall accuracy of proposed method.

The accuracy of proposed method has been calculated based on localization error during the packet transmission shown in Figure 7.

## 6. Conclusion

In this paper we develop a new novel strategy of discrete energy consumption model for wireless sensor network with the help of quasi and crude Monte Carlo method. In this model we assign the power in terms of discrete manner with the help of Bayesian network. By using discrete hidden Markov model each centralized node monitors the optimized energy of all sensor nodes in which every sensor state can be considered as the dynamic Bayesian network. Our esteemed simulation and experimental results show that the overall performance of our proposed method is better than the existing localization method of DQMCL and MCL methods.

## Figures and Tables

**Table 1:** Analysis of computational time with standard threshold values.

Novel strategy of proposed method				Discrete quasi Monte Carlo (DQMCL)				Monte Carlo localization (MCL)			
Number of power levels	Optimal threshold value	Computing time	Power mapping nodes	Number of power levels	Optimal threshold value	Computing time	Power mapping nodes	Number of power levels	Optimal threshold value	Computing time	Power mapping nodes
1	115	46	*n*_1_–*n*_6_	1	115	59	*n*_1_–*n*_6_	1	115	70	*n*_1_–*n*_6_
2	108	41	*n*_4_–*n*_9_	2	108	50	*n*_4_–*n*_9_	2	108	61	*n*_4_–*n*_9_
3	106	39	*n*_1_-*n*_2_	3	106	45	*n*_1_-*n*_2_	3	106	58	*n*_1_-*n*_2_
4	100	35	*n*_4_-*n*_5_	4	100	48	*n*_4_-*n*_5_	4	100	52	*n*_4_-*n*_5_
5	98	31	*n*_7_-*n*_8_	5	98	41	*n*_7_-*n*_8_	5	98	50	*n*_7_-*n*_8_
6	96	30	*n*_9_-*n*_8_	6s	96	35	*n*_9_-*n*_8_	6	96	47	*n*_9_-*n*_8_
7	94	28	*n*_6_-*n*_5_	7	94	34	*n*_6_-*n*_5_	7	94	44	*n*_6_-*n*_5_

**Table 2:** RSSI analysis with predefined value.

Novel strategy of proposed method				Discrete quasi Monte Carlo (DQMCL)				Monte Carlo localization (MCL)			
Predefined RSSI value (DB)	Achieved RSSI (DB)	Variations (DB)	Distance meter	Predefined RSSI value (DB)	Achieved RSSI (DB)	Variations (DB)	Distance meter	Predefined RSSI value (DB)	Achieved RSSI (DB)	Variations (DB)	Distance meter
−96	−95.5	−0.5	0.5	−96	−90	−6	0.5	−96	−88.2	−7.8	0.5
−95	−94.42	−0.58	0.9	−95	−92.5	−2.5	0.9	−95	−86.8	−8.2	0.9
−92	−90	−2	1	−92	−88.5	−3.5	1	−92	−80	−8	1
−89	−84	−5	1.8	−89	−80.23	−8.77	1.8	−89	−74.58	−14.42	1.8
−85	−82.22	−2.78	2	−85	−78.25	−6.75	2	−85	−70.48	−14.52	2
−80	−76.22	−3.78	3.5	−80	−75.6	−4.4	3.5	−80	−70.14	−9.86	3.5
−74	−71.12	2.88	4	−74	−69.12	−4.88	4	−74	−61.28	−12.72	4

**Table 3:** Discrete power levels of CC2430 [18] with power state variable.

Power level	Power state	Power (dbm) based on CC2430	Current (Ma) based on CC2430
1	0001	−0.2	29.9
2	0010	−0.5	31.1
3	0011	−0.7	29.1
4	0100	−0.9	28.1
5	0101	−1.2	29.7
6	0110	−1.5	26
7	0111	−2	26.6

**Table 4:** Experimental measurement of RSSI value with distance.

Distance in meter	Power level	RSSI (DBm)	Transmission packet ratio (out of 10)
0.5	1	−95.1	7
1	2	−94.2	7.2
1.5	3	−89	7.5
2	4	−84	8
2.5	5	−81.1	8.2
3	6	−76.1	8.4
3.5	7	−70	8.7

**Table 5:** Experimental measurement of localization error.

Power level	Localization error (%*R*)		
	Proposed method	DQMCL	MCL
2	44	50	55
4	40	45	50
6	39	42	45
